# 

**Published:** 1807-04

**Authors:** 


### To CORRESPONDENTS.

This Journal commenced with Vaccination, and to the present time it has never lost sight of its progress. Ignorance has been enlightened by information on that important subject; prejudice has been combated by facts and reasoning; misrepresentation and falsehood have been detected and exposed with patience and diligence; even malicious calumny has been made to recoil on its own authors. Having therefore at length witnessed the triumph of humanity and liberality over apathy, disappointed selfishness, and malignant envy; we propose to omit, or greatly abridge, all future communications on that subject. Another weighty reason for this conduct is furnished by the Report of the Royal College of Physicians, made in obedience to his Majesty's command, which doubtless will set the question at rest, with all the disinterested, candid part of the public, and even impose silence upon obstinacy and prejudice.

The valuable Communication of Mr. Thomas Young, (late of Coleman Street, now of Christopher Street, Finsbury Square) "on the Organization of the Iris, and on Artificial Pupils," will appear in our next Number, accompanied with Engravings.

